# The Reliability and Validity of a Modified Squat Test to Predict Cardiopulmonary Fitness in Healthy Older Men

**DOI:** 10.1155/2018/4863454

**Published:** 2018-01-02

**Authors:** Chiu-Ping Yeh, Hsien-Cheng Huang, Yaju Chang, Ming-De Chen, Miaoju Hsu

**Affiliations:** ^1^Department of Physical Education, National Taiwan Normal University, Taipei, Taiwan; ^2^Physical Educational Office, National Kaohsiung University of Applied Sciences, Kaohsiung, Taiwan; ^3^Section of Cardiology, Department of Internal Medicine, Taipei City Hospital, Yangming Branch, Taipei, Taiwan; ^4^Department of Physical Therapy and Graduate Institute of Rehabilitation Science, Chang Gung University, Taoyuan, Taiwan; ^5^Department of Occupational Therapy, College of Health Science, Kaohsiung Medical University, Kaohsiung, Taiwan; ^6^Department of Physical Therapy, College of Health Science, Kaohsiung Medical University, Kaohsiung, Taiwan; ^7^Department of Rehabilitation, Kaohsiung Medical University Hospital, Kaohsiung, Taiwan

## Abstract

**Background:**

Shortcomings are noted in currently available cardiopulmonary field tests for the older adult and thus relevant research is still ongoing.

**Purpose:**

The purpose of this study was to investigate the reliability and validity of a modified squat test and to establish a regression model for predicting aerobic fitness in the older adult.

**Methods:**

Twenty-five healthy men aged 60 to 75 years completed this study. Each subject performed two modified squat tests with a prototype testing equipment and a maximal exercise test to determine maximal oxygen consumption. Recovery heart rates (HR) (0~30, 60~90, and 120~150 seconds) were measured following the modified squat tests. The fitness indexes included the sum of recovery HR, recovery HR index, age-adjusted recovery HR index, and immediate HR.

**Results:**

The results revealed that the age-adjusted recovery HR index fitness had the highest intraclass correlation coefficients (ICC) of 0.9 and Pearson's correlation coefficients of 0.71, which suggested the modified squat test can reasonably assess cardiopulmonary fitness for the older adult. The regression equation for estimating aerobic power was ${\dot{V}O}_{2}max$ = 16.781 + 16.732 × (age-adjusted recovery HR index) + 0.02467 × (physical activity level).

**Conclusion:**

The modified squat test is a valid and reliable field test and thus can be an option to assess the cardiopulmonary fitness level of healthy older men in clinics or communities.

## 1. Introduction

Cardiopulmonary fitness, which is most accurately presented by maximal oxygen uptake (${\dot{V}O}_{2}max$), is related to mortality in older adults. Laboratory maximal exercise tests with a treadmill or an ergometer can precisely evaluate ${\dot{V}O}_{2}max$, which is not always accessible due to expensive equipment and trained personnel being required [1, 2]. For practical purposes, field tests are developed and used to measure cardiopulmonary fitness. Based on the characteristics of the older adult, the common test modes of field tests are stepping and walking tests.

A three-minute stepping test is widely used to predict cardiopulmonary fitness in that heart rate (HR) responses immediately or during recovery after the stepping test are recorded and calculated. Different stepping frequency, bench height, test duration, the number of stages, and the scoring method have been developed for particular populations. For example, Petrella et al. [3] established a self-paced step test for the community-dwelling older adult, which is implemented at 40 cm height and requires stepping 20 times at different paces. A high correlation coefficient of 0.75~0.94 is found between the observed and the predicted maximal oxygen consumption (${\dot{V}O}_{2}max$). Though the self-paced step test appears to accurately estimate cardiopulmonary fitness in older adults, it is argued that stepping movements might cause orthopedics problems in knees as well as increase the fall risk to mobility-limited older adults [4]. Rikli et al. [5, 6] developed a two-minute self-paced test involving stepping in place, which is safer for the older adult. However, the relationship between this two-minute step-in-place test and maximal oxygen consumption is not established.

The walking test employs functional movements in nature and is easy to conduct. Particularly, the walking test is much safer than the stepping test and thus is more commonly used in senior people. Six-minute walking test and Rockport fitness walking test can reasonably predict cardiopulmonary fitness in the elderly [7, 8] while shortcomings are noted. Numerical studies have demonstrated that the 6 min walking test is not appropriate to evaluate changes in cardiorespiratory fitness in healthy older men who received endurance training for 24 weeks [9–12]. Moreover, a spacious walkway is needed. Therefore, research on developing new field tests of cardiopulmonary fitness for the older adult is still ongoing.

Inoue and Nakao developed a cardiopulmonary fitness test, a squat test, that is simple to administer in a confined space with minimum apparatus [13]. Participants should repeat squatting 30 times per min by bending of the legs until the hips meet with the heels. A significant correlation (*r* = 0.92 for women, *r* = 0.82 for men, *p* < .001) between ${\dot{V}O}_{2}max$ and the fitness index of the squat test has been found in young adults. Considering difficulties to fully squat down in the older adult and the heavy loading of the knee joint during the squatting activity due to the nature of the movement, we modified the original squat test from full squatting to half-squatting in order to minimize possible injuries to knee joints. The purpose of this study was to evaluate the reliability and validity of the modified squat test and to construct a model for the estimation of aerobic fitness based on the modified squat test performance.

## 2. Method

### 2.1. Participants

Thirty-three healthy older subjects between the ages of 60 and 75 years participated in this study. Exclusion criteria included cardiovascular disease, metabolic disease, pulmonary disease, mental problems, and severe orthopedic diseases of the lower extremity. Informed consent was obtained from all participants. To assess potential risks of performing a maximal exercise test, the Physical Activity Readiness Questionnaire (PAR-Q) was administered and the resting 12-lead EKG was conducted and screened by a cardiologist. Before testing, body weight, height, body fat (TANITA, Taiwan), and Physical Activity Scale for the Elderly (PASE) score were recorded.

### 2.2. Experimental Protocol

All participants had to complete a maximal exercise test and two modified squat tests. The subject performed the first modified squat test, followed by the second modified squat test with oxygen consumption measured and then a maximal exercise test. An adequate rest was provided between the tests in order to allow heart rate and blood pressure to be returned to the level of baseline, which was defined as within 5 beats/minutes for heart rate and 5 mmHg for blood pressure.

### 2.3. Maximal Exercise Test

Aerobic capacity was measured by performing a treadmill exercise test using the Cornell-modified Bruce treadmill exercise protocol (Table 1). The protocol, which was used to determine the cardiopulmonary fitness of the elderly, consists of 2-min stages, beginning with 0 stage at 1.7 mph and 0% grade, gradually increasing intensity to stage 5 of the Bruce protocol [14, 15]. The 12–lead EKG was monitored during the test as well as the recovery stage. Heart rate was recorded every minute, and blood pressure and Borg's rating of perceived exertion (RPE) on a scale of 6–20 were assessed every 2 minutes. Respiratory gas samples were analyzed breath-by-breath using a portable metabolic system (K4b^2^, COSMED, Rome, Italy). The test was terminated based on American College of Sports Medicine (ACSM) guidelines for conducting a maximal exercise test [16, 17], with the age-predicted maximum heart rate calculated as (208 − 0.7 × age) [18] and respiratory exchange ratio (RER) adjusted for 1.00 [19]. Aerobic power (${\dot{V}O}_{2}max$), HR_max_, maximal blood pressure, rating of perceived exertion (RPE) based on a 6–20 points' Borg Scale, and the time of the exercise test were obtained.

### 2.4. Modified Squat Test

**Figure 1:**
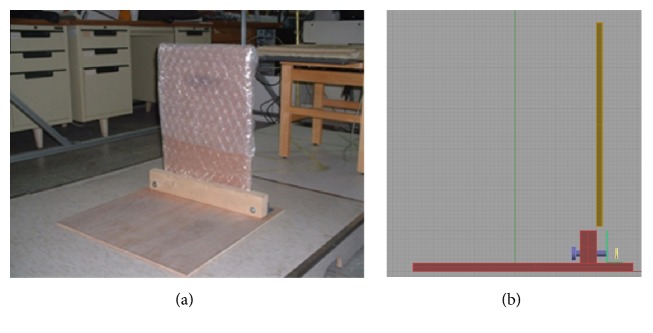
Custom-made lightweight equipment. (a) Assembled testing device. (b) A diagram from a lateral view showing the upright part of the device can be detached from the horizontal part.

**Figure 2:**
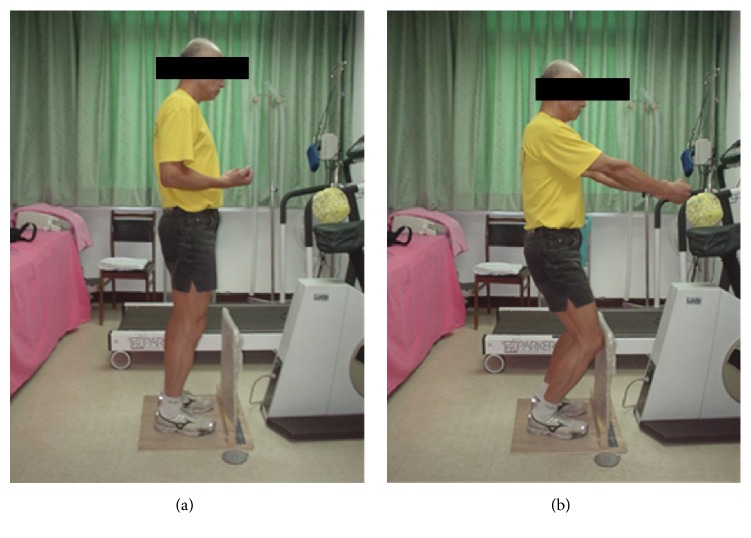
Movements of the modified squat test. (a) Starting position. (b) Ending position.

A custom-made lightweight equipment platform (Figure 1(a)) was developed to conduct the modified squat test. The vertical part of the testing equipment (Figure 1(b)) could be detached from the horizontal part so that the equipment could be easily carried. When performing the modified squat test, the subject started at a standing position with his elbows 90° flexed at the sides of the waist (Figure 2(a)), followed by squatting down to 45° knee flexion with both arms pushed out at the same time (Figure 2(b)), and then returned to the starting position (Figure 2(a)). The subject repeated the above-mentioned sequences at a rate of 104 cycles/min for 3 minutes, using a metronome. Recovery heart rates (HR) (0~30, 60~90, and 120~150 seconds) were counted following the modified squat test with using a stethoscope. Blood pressure and RPE were recorded at the end of the test. To compare with previous studies, we calculated several fitness indexes including immediate HR, the sum of recovery HR, and recovery HR index (18000/((HR_0~30_ + HR_60~90_ + HR_120~150_) × 2)). In addition, we developed a new index calculated as (recovery HR index/age). The indexes obtained from the modified squat test were determined as follows.

#### 2.4.1. Immediate HR

After the termination of the modified squat test, heart rate measured from Polar was recorded immediately.

#### 2.4.2. The Sum of Recovery HR

When the modified squat test was finished, cumulative heart beats were counted during 0~30, 60~90, and 120~150 seconds. The total number of recovery pulse counts was calculated as the sum of recovery HR.

#### 2.4.3. Recovery HR Index

The fitness index was revised from the equation of Harvard step test [20]:

(1) Recovery HR index=test duration of modified squat test second×100(the sum of recovery HR)×2.

#### 2.4.4. Age-Adjusted Recovery Heart Rate

The fitness index was calculated according to the following equation:

(2) Age-adjusted recovery heart rate=recovery HR index age.

### 2.5. Statistical Analysis

Test-retest reliability for the modified squat test was established by determining the intraclass correlation coefficient (ICC). The ICC values greater than 0.75 were considered as good reliability, those between 0.5 and 0.75 as moderate reliability, and those below 0.5 as poor reliability [21]. Pearson correlation analysis was used to evaluate the correlation between the fitness indexes of the modified squat test and the maximal exercise test performance. Correlation coefficients (*r*) higher than 0.6 were defined as high correlations, those between 0.3 and 0.6 as moderate correlations, and those under 0.3 as poor correlations [21]. To predict ${\dot{V}O}_{2}max$ from the best fitness index, a stepwise multiple regression analysis was performed with physical activity level and physiological and anthropometric data (age, resting HR, height, weight, BMI, and percent of body fat) as independent variables.

## 3. Results

### 3.1. Participant Characteristics

Thirty-three male participants aged 60 to 75 years were recruited. Eight of these participants did not complete the maximal exercise test due to cardiac and/or balance problems and were excluded for data analysis. The baseline characteristics of the remaining 25 participants analyzed are presented in Table 2.

### 3.2. Maximal Graded Exercise Test

Physiological responses of the participant to the maximal exercise test are shown in Table 3. Twenty-five subjects completed the maximal exercise test without any abnormal ECGs or complications. The ${\dot{V}O}_{2}$ and RER at baseline were 4.86 ± 1.03 (ml/kg/min) and 0.76 ± 0.10, respectively. The ${\dot{V}O}_{2}$ and RER at maximal efforts reached 35.82 ± 4.44 (ml/kg/min) and 1.05 ± 0.11, respectively. The time to volitional exhaustion was within an optimal exercise time of 8 to 12 minutes as suggested by ACSM.

### 3.3. The Modified Squat Test

Table 4 presents the result of the two modified squat tests. As shown in Table 4, the intensity for the modified squat test was 20.72 ± 3.60 ml/kg/min, corresponding to 58.22 ± 10.00% of ${\dot{V}O}_{2}max$ and 80.98 ± 10.92% of age-predicted maximal HR.

### 3.4. Reliability of Squat Test

All the modified squat test fitness indexes showed high test-retest reliabilities, with ICC values ranging from 0.77 to 0.90. As shown in Table 5, age-adjusted recovery HR index had the highest ICC value (0.90) among the four squat test indexes.

### 3.5. Validity of the Modified Squat Test

As shown in Table 6, a significant negative correlation was seen between the sum of recovery HR and ${\dot{V}O}_{2}max$, whereas significant positive correlations between the recovery HR index and age-adjusted recovery index and ${\dot{V}O}_{2}max$ were found. Age-adjusted recovery HR index had the highest correlation with ${\dot{V}O}_{2}max$.

### 3.6. Prediction of ${\dot{V}O}_{\text{2}}max$ Equation

Table 7 presents the result of the stepwise multiple regression analysis for prediction of ${\dot{V}O}_{2}max$. Age-adjusted recovery HR index and physical activity level were strongly correlated with ${\dot{V}O}_{2}max$ and accounted for 63% of the variance. The prediction equation for ${\dot{V}O}_{2}max$ from the modified squat test was

(3) V˙O2max=16.781+16.732×Age-adjusted recovery HR index+0.02467×physical activity level.

## 4. Discussion

Cardiopulmonary fitness is associated with risks of cardiovascular diseases [22–24]. With aging, maximal oxygen consumption declines at the rate of 1% per year [25]. Regular exercise has been considered to be a safe and effective strategy to delay the aging process. Evaluation of cardiopulmonary fitness is essential to assure that a safe exercise prescription is implemented for older people [26]. Considering the nature of physical characteristics of older individuals, we modified a squat test, which was originally designed for young healthy adults.

### 4.1. Reliability

The ICC analyses on the fitness indexes (the immediate HR, sum of recovery HR, recovery HR index, and age-adjusted recovery HR index) of the modified squat test revealed that the ICCs ranged from 0.77 to 0.90, suggesting high reliabilities according to the definition of reliability level proposed by Portney and Watkins [21]. Age-adjusted recovery HR index had the highest ICC among the four modified squat test fitness indexes, showing the best reliability. On the contrary, the immediate HR yielded the lowest ICC value (0.77). The older adult has greater variations in physiological responses to an exercise and slower adaptations to an exercise, which contributes to the lower ICC seen in the immediate HR fitness index.

Petrella et al. investigated the validity of the self-paced step test in older adults while the reliability was not conducted [3]. Kervio et al. assessed the reliability of the 6-minute walk test in 12 healthy old individuals [27]. In their study, six trials of the 6-minute walk test were performed. However, only the coefficient of variation (CV) instead of ICCs was reported. Fenstermaker et al. investigated the test-retest ICC reliability of the Rockport walking test on the fitness indexes of walking time, HR, and estimated ${\dot{V}O}_{2}max$ [9]. The ICC values of Rockport walking fitness indexes ranged from 0.67 to 0.71. In our study, a high ICC of 0.90 for age-adjusted recovery HR index suggests that the reliability of the modified squat test is comparable or superior to the above-mentioned fitness tests.

### 4.2. Validity

Previous studies have developed several aerobic fitness tests for old individuals. Rikli and Jones investigated the validity of the 6 min walk test in older individuals by correlating the treadmill performance time reaching 85% of age-predicted maximal heart rate with walking distance and found a high correlation of 0.78 [11]. The two-minute step-in-place test had a correlation coefficient (*r*) of 0.74 between number of steps and treadmill time [5, 6]. Petrella et al. reported the validity for the self-paced step test in healthy older individuals in that a moderate to high correlation coefficient of −0.58~−0.74 between step time and ${\dot{V}O}_{2}max$ and a poor correlation coefficient of 0.03~−0.11 for HR were found [3].

Though those commonly seen aerobic fitness tests have acceptable validity, there are some shortcomings. For example, the 6 min walk test needs a spacious testing environment. The self-paced step test used a step with 40 cm height, which may predispose older individuals to increased risk of falls during testing. In our study, moderate to high correlations between fitness indexes and ${\dot{V}O}_{2}max$, with the highest correlation of 0.7 seen in age-adjusted recovery HR index, suggested the modified squat test is a valid fitness test in the older individual and comparable to or even superior to other fitness field tests reported in previous studies. The modified squat test is convenient, has low cost, is safe to administer, and requires limited space and thus may be another option for assessing aerobic fitness for old healthy individuals.

Recovery HR after exercise has been found to be correlated with the fitness level and mortality in old individuals [28, 29]. The immediate HR fitness index had the worst validity among the fitness indexes. One contributory factor might be due to the aged autonomic nervous system. Immediate HR recovery is primarily a function of reactivation of the parasympathetic nervous system, while later recovery of HR is associated with gradual withdrawal of the sympathetic nervous system [30, 31]. Aging results in slower adaptations of the autonomic nervous system to the termination of exercise [32, 33]. In other words, it takes longer for older adults to recover their HR to the baseline. Therefore, fitness indexes using several recovery HRs would be more representative of the fitness level of the older individual.

### 4.3. Prediction of Equation

Kervio et al. reported that predicted ${\dot{V}O}_{2}max$ equation for older individuals from the 6-minute walk test parameters (distance, heart rate) and anthropometric value (age, weight, and height) accounted for 94% of the variance in ${\dot{V}O}_{2}max$, with a small subject number of 12 [27]. In the self-paced step test, stepping time, heart rate, age, BMI, and O_2_ pulse were significantly associated with ${\dot{V}O}_{2}max$ and were chosen to establish the predictive formula, which can explain 72% to 86% of variance [3]. The correlation of the predicted ${\dot{V}O}_{2}max$ of the self-paced step test and the measured ${\dot{V}O}_{2}max$ was from 0.88 and 0.90 for low-fitness men and women and 0.83 and 0.94 for high-fitness men and women. In our study, the age-adjusted recovery HR fitness index had the highest validity among four fitness indexes calculated and thus was chosen to develop a prediction equation for aerobic power of the older individual. Factors which might affect ${\dot{V}O}_{2}max$ value such as age, resting HR, height, weight, body fat, and physical activity were taken into account for regression analysis to establish the prediction of ${\dot{V}O}_{2}max$. The predicted ${\dot{V}O}_{2}max$ using this model was highly correlated (*r* = 0.79) with measured ${\dot{V}O}_{2}max$ from the maximal exercise test. The predictive model based on age-adjusted recovery HR and physical activity explained 63% of the variances in ${\dot{V}O}_{2}max$. The variance explained by the prediction equation in our study appears to be lower than those in the 6 min walk test and the self-paced step test. However, only 12 subjects were recruited to develop the prediction equation from the 6 min walk test in Kervio et al. study. In the prediction model for the self-paced step test, O_2_ pulse was used as one of predictive parameters. Petrella et al. indicated that O_2_ pulse from the self-paced step test was strongly associated with ${\dot{V}O}_{2}max$ and improved the percentage variance to be explained in the prediction of ${\dot{V}O}_{2}max$ [3]. For clinical practice purposes, we did not include O_2_ pulse in our model, which might contribute to the discrepancy. In addition, previous research stated that body composition, such as BMI, weight, or body fat, is a factor in the predictive equation of ${\dot{V}O}_{2}max$ [3, 9, 27]. However, in our study, body composition was excluded in the regression analyses. A small sample size and homogeneous body fat in our subjects might be contributory factors.

### 4.4. Suggestions for Future Applications of the Modified Squat Test

The exercise intensity for the modified squat test is approximately 60% of ${\dot{V}O}_{2}max$ and 81% of age-predicted maximal HR, corresponding to a moderate exercise workload. No discomfort or injuries during the testing were reported. The modified squat test is submaximal while appearing to be able to elicit substantiate physiological exercise responses to allow one's maximal aerobic power to be assessed safely and accurately. Moreover, it is interesting to note that most of the subjects expressed that they would practice the modified squat test as an exercise afterwards, suggesting the continuous movement of the modified squat test might potentially be developed as an interesting form of exercise. To serve this purpose, the prototype testing device used in this study should be further developed to provide adjustments of exercise workloads as well as feedback of exercise intensity. The prototype testing device could be manufactured with sensors monitoring HR and with a device indicating different rates of half-squatting. In addition, an electronic goniometer could well be integrated into the testing system to indicate the appropriate angle of squatting.

There are several limitations in this study. First, the sample size of this study was small. In addition, most of the subjects tended to be young older adults (aged < 70 years). Therefore, a larger sample size with individuals aged 70 years and beyond is required in future studies to enhance the application of the ${\dot{V}O}_{2}max$ prediction equation determined from the modified squat test. Second, the subjects of this study were all males. Whether the modified squat test is valid for healthy older females still needs to be confirmed.

## 5. Conclusions

The results reveal that the modified squat test is valid and reliable and can be an option for evaluating the fitness level in healthy elderly men in clinics or communities. The best index is age-adjusted recovery heart rate. The predicted equation for ${\dot{V}O}_{2}max$ is 16.781 + 16.732 × age-adjusted recovery HR + 0.02467 × physical activity level (score of PASE questionnaire).

## Figures and Tables

**Table 1:** Cornell-Modified Bruce Protocol.

Stage	Min	Speed (mph)	Grade (%)
0.0	2	1.7	0
0.5	4	1.7	5
1.0	6	1.7	10
1.5	8	2.1	11
2.0	10	2.5	12
2.5	12	3.0	13
3.0	14	3.4	14
3.5	16	3.8	15
4.0	18	4.2	16
4.5	20	4.6	17
5.0	22	5.0	18

**Table 2:** Physical characteristics and baseline physiological parameters for the subject (*N* = 25).

	Mean	SD
Age (years)	65.16	4.90
Weight (kg)	66.11	8.58
Height (cm)	166.52	5.21
BMI (kg/m^2^)	23.81	2.59
Body fat (%)	20.57	5.73
Heart rate rest (beats/min)	73.80	11.69
Systolic blood pressure (mmHg)	128	14.59
Diastolic blood pressure (mmHg)	81.28	9.34
Physical Activity Scale for the Elderly	137.07	64.81

SD: standard deviation.

**Table 3:** Physiological responses to maximal exercise test (*N* = 25).

	Mean	SD
${\dot{V}O}_{2}max$ (ml/kg/min)	35.82	4.44
RER	1.05	0.11
Time to exhaustion (min)	12.80	2.00
HRmax (beats/min)	165.12	8.25
Systolic blood pressure (mmHg)	196.75	18.88
Diastolic blood pressure (mmHg)	88.00	10.49
RPE	17.28	1.24

SD: standard deviation.

**Table 4:** Parameters of the modified squat test (*N* = 25).

	S1	S2
Immediate HR (beats/min)	131.40 ± 17.85	134.04 ± 18.63
Recovery HR_0–30 s (beats)	51.16 ± 9.59	58.36 ± 9.63
Recovery HR_60–90 s (beats)	47.80 ± 8.27	48.60 ± 9.57
Recovery HR_120–150 s (beats)	44.84 ± 8.01	45.96 ± 9.17
Sum of recovery HR (beats)	149.80 ± 24.79	152.92 ± 27.45
Recovery HR index	61.92 ± 11.81	60.70 ± 10.85
Age-adjusted recovery HR index	0.96 ± 0.21	0.94 ± 0.18
$\dot{V}{\text{O}}_{2}$ (ml/kg/min)	—	20.72 ± 3.60
Percentage of ${\dot{V}O}_{2}max$ (%)	—	58.22 ± 10.00
Percentage of age-predicted HR_max_ (%)	80.93 ± 10.88	82.53 ± 11.05
SBP (mmHg)	167.11 ± 17.27	164.27 ± 22.48
DBP (mmHg)	95.78 ± 8.21	88.18 ± 11.43
RPE	12.20 ± 1.44	12.48 ± 1.81

S1: the first modified squat test; S2: the second modified squat test with oxygen consumption measurement; —: not available.

**Table 5:** Test-retest reliability of the modified squat test (*N* = 25).

Index	Immediate HR (beats)	Sum of recovery HR (beats)	Recovery HR index	Age-adjusted recovery HR index
ICC	0.77	0.88	0.87	0.90

**Table 6:** Correlations between the squat test indexes and ${\dot{V}O}_{2}max$ (*n* = 25).

	S1	S2
Immediate HR (beats/min)	−0.52^*∗∗*^	−0.51^*∗*^
Sum of recovery HR (beats)	−0.64^*∗∗*^	−0.60^*∗∗*^
Recovery HR index	0.68^*∗∗*^	0.64^*∗∗*^
Age-adjusted recovery HR index	0.70^*∗∗*^	0.71^*∗∗*^

^*∗∗*^ *p* < .001; ^*∗*^*p* < .05; S1: the first modified squat test; S2: the second modified squat test with oxygen consumption measurement.

**Table 7:** The regression model for the modified squat test to predict ${\dot{V}O}_{2}max$.

	Regression coefficient (*B*)	SE	Standardized regression coefficients (*β*)	*p* value
Age-adjusted recovery HR index	16.732	3.149	0.497	.000
Physical activity level	0.02467	0.009	0.130	.000
Constant	16.781	3.203		.011
*R* ^2^	0.63			
Adjusted *R*^2^	0.59			

SE: standard error; *R*^2^: coefficient of determination.
